# The modified Yi qi decoction protects cardiac ischemia-reperfusion induced injury in rats

**DOI:** 10.1186/s12906-017-1829-6

**Published:** 2017-06-21

**Authors:** Xiao Yu, Xiao-Dong Zhao, Rong-Qi Bao, Jia-Yu Yu, Guo-Xing Zhang, Jing-Wei Chen

**Affiliations:** 1 Laboratory of Cancer Molecular Genetics, Medical College of Soochow University, 199 Ren-Ai Road, Dushu Lake Campus, Suzhou Industrial Park, Suzhou, 215123 People’s Republic of China; 2 Department of Physiology, Medical College of Soochow University, 199 Ren-Ai Road, Dushu Lake Campus, Suzhou Industrial Park, Suzhou, 215123 People’s Republic of China; 3 Department of Internal Medicine, the Affiliated Suzhou Chinese Traditional Medicine Hospital, Nanjing University of Chinese Medicine, 18 Yang-Su Road, Suzhou, 215003 People’s Republic of China

**Keywords:** Modified Yi qi decoction (MYQ), Cardiac ischemia-reperfusion, Apoptosis, Calcium handling proteins, Autophagy

## Abstract

**Background:**

To investigate the effects and involved mechanisms of the modified Yi Qi decoction (MYQ) in cardiac ischemia-reperfusion (IR) induced injury.

**Methods:**

Male Sprague-Dawley rats were subjected to a 30-min coronary arterial occlusion followed by reperfusion, low or high dose decoction of MYQ was administrated orally for 1 week or 1 month.

**Results:**

Both in 1 week and 1 month IR rat groups, cardiac function indexes were significantly impaired compared with sham group rats, accompanied with higher ratio of infarct size to risk size, decreased expressions of sodium calcium exchanger (NCX1) and sarcoplasmic reticulum Ca^2+^-ATPase (Serca2a), and different expressions of autophagic proteins, Beclin-1 and LC3. Treatment with MYQ (low or high dose) for 1 week showed no marked beneficial effects on cardiac function and cardiac injury (ratio of infarct size to risk size), although expressions of anti-apoptotic protein, Bcl-2, NCX1 and Serca2a were increased. Treatment with MYQ (low or high dose) for 1 month showed significantly improved effects on cardiac function and cardiac injury (ratio of infarct size to risk size), accompanied with increase of Bcl-2, NCX1 and Serca2a expressions, and decrease of Bax (a pro-apoptotic protein) and Beclin-1 expressions.

**Conclusions:**

The results show that MYQ have potential therapeutic effects on IR-induced cardiac injury, which may be through regulation of apoptotic proteins, cytosolic Ca^2+^ handling proteins and autophagic proteins signal pathways.

## Background

Heart failure is still a major public health problem worldwide with high mortality and morbidity [1, 2]. It can be the result of various diseases, coronary artery disease and subsequent myocardial infarction is a common cause of heart failure. The rapid energy depletion suppresses metabolic activity and leads to the induction of cardiomyocytes cell death pathways. Reperfusion ameliorates the extent of cell death, but in turn it invokes lethal reperfusion injury, which may proceed via necrosis, apoptosis and autophagy [3]. The mechanisms responsible for ischemia-reperfusion (I/R) injury have been widely investigated, calcium overload, excessive production of reactive oxygen species (ROS), and the release of inflammatory factors are the major causative factors of cardiac I/R injury [4–6]. A large number of strategies has been proposed to improve cardiac function related to those mechanisms, however, the clinic outcomes are still not satisfied.

Chinese herbal medicine, especially combined herbal formulation, has been widely applied for cardiovascular diseases for hundreds of years. Possible mechanisms have been well reviewed [7], including: (1) anti-oxidation, (2) anti-inflammation, (3) anti-apoptosis, (4) protection of mitochondria function, (5) promoting angiogenesis, (6) increasing bone marrow derived stem cells migration, (7) inhibiting Ca^2+^ overload. According to the theory of Traditional Chinese Medicine, the principal mechanism of cardiovascular disease is considered to be a disorder or deficiency of Qi (energy) and a disorder of the circulation (blood stasis) which lead to severe pain and even death. Therefore, the main aims of Chinese herbs and herbal formulations in cardiovascular diseases treatment are to regulate or replenish Qi, and/or to unblock circulation or resolve blood stasis. However, studies of Chinese decoctions or formulations in treatment of cardiovascular diseases are relatively scarce, although decoction and formulations are the main forms of therapy in Traditional Chinese Medicine practice.

Wu Meng Therapy has long history for more than 2500 years. Xi Feng-Lin, a grandmaster of Chinese Medicine, established ten principles for treatment of coronary disease according to *Synopsis of Preions of the Golden Chamber* written by Zhang Zhong-Jing of Eastern Han Dynasty (150–219), its main concept is treatment of heart diseases should combine with improvement of heart, spleen and stomach functions. The modified Yi Qi decoction (MYQ) consists of five herbs including *Milkvetch Root, Semen Pharbitidis, Cinnamom Twig, Fructus Amomi, and White Peony Root. Milkvetch Root*, has been used for more over 2000 years in China, can strengthen immune function, protect liver, promote urination, resist aging and stress, reduce blood pressure and extensively resist bacterium [8–12]. *Semen Pharbitidis* has anti-tumor and anti-metastasis effects in Lewis lung cancer [13]. Recently, it was also reported that a Traditional Chinese Medicine Herbal Ointment, mainly containing *Semen Pharbitidis* could reduce malignant pleural effusion [14]. *Cinnamom Twig* also has been widely used in traditional Chinese medicine for the treatment of endometriosis-related symptomatic discomfort [15], uterine fibroid [16]. Recently, anti-hyperglycemic and antioxidant activities of twig extract from Cinnamomum osmophloeum also have been reported [17]. *Fructus Amomi* also has hypoglycemic, antioxidative and anti-allergic inflammatory activities [18–20]. *White Peony Root* is a component herb of many traditional formulae, such as Siwu-Tang, which has been widely used in treating palpitation, dysmenorrhea, chronic inflammation, anemia, depression, diabetic peripheral neuropathic pain and acute radiation-induced esophagitis [21–24].

Importantly, Yi Qi decoction has been widely applied in ancient Chinese patients and recent scientific researches supporting its beneficial effects. Yin et al. demonstrated that a Yi Qi decoction, Shu-Mai-Tang could attenuate TNFα-induced myocardial fibrosis in myocardial ischemia rats [25]. Mark et al. also reported that another Yi Qi decoction, Dang-Gui Buxue Tang, possesses a more potent cardioprotective effect than it’s extracts from different component herbs and enhances glutathione status in rat heart mitochondria and erythrocytes [26]. Other Yi Qi decoctions, Shu-mai-tang, Dan-Chuan-Hong also have beneficial effects on angiogenesis, arteriogenesis, anti-apoptosis and improvement of cardiac function in rats with myocardial ischemia [27, 28]. Recently, Danshen-Gegen decoction, also belonging to Yi Qi decoction, has been reported to protect the myocardium against I/R injury via the redox-sensitive PKCε/mKATP pathway in rats [29], and protect against hypoxia/reoxygenation-induced apoptosis by inhibiting mitochondrial permeability transition via the redox-sensitive ERK/Nrf2 and PKCε/mKATP pathways in H9c2 cardiomyocytes [30]. These above-mentioned litterateurs suggest that Yi Qi decoction may protect cardiac against I/R injury, however, different compositions of decoction may have different effects and via different signal pathways. Therefore, in the present study we carry out our investigation into the effects and mechanisms of Yi Qi decoction with modification (MYQ) on cardiac I/R induced injury.

In the present study, we applied rat I/R model and treated with different dosages of MYQ after reperfusion for 1 week or 1 month. Cardiac performance after one-week and one-month reperfusion is examined to testify the therapeutic effects of MYQ. Furthermore, mechanisms of therapeutic effects of MYQ are investigated by detecting the expressions of apoptotic signal pathway proteins, calcium handling proteins, autophagic signal pathway proteins.

## Methods

### Preparation of MYQ

MYQ was prepared from five dried raw materials (Table 1) purchased from Suzhou Chinese Traditional Medicine Hospital (Suzhou, China) and authenticated by a pharmacist of traditional Chinese medicine in Suzhou Chinese Traditional Medicine Hospital and all voucher specimens are deposited in the herbarium center of Suzhou Chinese Traditional Medicine Hospital. All raw materials were extracted by boiling in distilled water (about 6-fold the weight of the mixture) at 100 °C for 20 min and then filtered. The filtrates were stored at −80 °C for further application.

**Table 1:** Composition of MYQ

Chinse name	Latin name	English name	Amount (g)	Place of origin
Huang Qi	*Astragalus membranaceus var. mongholicus (Bunge)* P.K. Hsiao	Milkvetch Root	30	Inner Mongolia, China
Hei Chou	*Pharbitis nil* (L.) Choisy	Semen Pharbitidis	10	Jiangsu, China
Gui Zhi	*Cinnamomum cassia* (L.) J. Presl	Cinnamom Twig	10	Guangdong, China
Sha Ren	*Amomum villosum* Lour.	Fructus Amomi	5	Guangdong, China
Bai Shao	*Paeonia lactiflora* Pall.	White Peony Root	10	Zhejian, China

### Experimental animals

Ten-week-old male Sprague-Dawley rats were purchased from Shanghai Laboratory Animal Center. Rats were housed under optimal conditions with standard hygiene, kept at a temperature of 25 °C with a 12/12 light/dark cycle, fed with standard rat chow and water ad libitum. The experiments were performed in according with the National Institutes of Health Guidelines for the Use of Laboratory Animals (NIH, publication number 85–23, revised 1996.), which were approved by and performed according to guidelines for the care and use of animals established by Soochow University of Animal Care and Use Committee. All surgery was performed under sodium pentobarbital anesthesia, and all efforts were made to minimize animal suffering.

### Myocardial I/R model

The I/R model was performed as our previous report [31]. Briefly, rats were anesthetized with sodium pentobarbital (50 mg/kg i.p.), cardiac I/R was performed by exposing the heart at the fifth intercostal space followed by a slipknot (6–0 silk) below the left descending coronary artery. Regional left ventricular ischemia was performed via occlusion of the coronary artery by clamping it together with the propylene tube. After 30 min of ischemia, the slipknot was released and ischemic part was subjected to reperfusion. Two hours later, rats were treated with or without MYQ solution for 1 ml (low dose, equals to clinical patient’s dosage calculated according to body surface area) or 4 ml (high dose) by gastric feeding. Then, everyday rats were treated three times with above-mentioned dosages. Sham group is without occlusion (*n* = 10), low dose treatment of I/R rats lasts for 1 week (*n* = 10) or 1 month (*n* = 10), high dose treatment of I/R rats also lasts for 1 week (*n* = 9) or 1 month (*n* = 9).

### Cardiac function measurements

One week or 1 month later after I/R, rats were anesthetized with sodium pentobarbital (50 mg/kg i.p.), and hemodynamic parameters were measured using a heart performance analysis system (ALCBIO, Shanghai Alcott Biotech CO., LTD.). The left femoral artery and right common carotid artery were isolated. A polystyrene PE-50 catheter was inserted into the left ventricle via right common carotid artery, with the other end connected to the analysis system. The major parameters of cardiac function were derived or calculated from the continuously obtained pressure signal, including systolic arterial pressure (SAP), the rate of maximum positive and negative left ventricular pressure development (± LVdp/dt max), and the left ventricular end-diastolic pressure (LVEDP), etc.

### Measurement of ratio of myocardial infarct area to area at risk

After rat’s cardiac function was measured under anesthetized condition with sodium pentobarbital (50 mg/kg i.p.), rat hearts were excised immediately and perfused with Evans blue (1%, 4 ml) via the coronary artery under ligation of the left descending coronary artery with the remained sutures. Hearts were traversely cut into 1–2 mm slices along the ligation point, placed in 1.25% 2,3,5-triphenyltetrazolium chloride (TTC; Sigma, USA) solution in PBS, incubate for 10 min at 37 °C. The ischemic regions (area at risk, AAR) and the infarct area (white area is not stained by TTC) were recorded by digital camera, and blue area (stained by Evans blue; non-ischemic area) were analyzed with a digital imaging system (NIH image software). The ratio of myocardial infarct area to area-at-risk (AAR) was calculated.

### Western blot analysis

Myocardial tissues (AAR tissue) were homogenized with RIPA buffer (50 mm Tris, ph 7.0, 150 mM NaCl, 1% Triton-X-100) containing phenylmenthanesulfonyl fluoride (R&D Systems Inc., Minneapolis, US). Homogenates were centrifuged at 12,000×g for 10 min at 4 °C. Cell protein were separated by SDS-PAGE and transferred to PVDF membranes (Hybond TM-ECL; Amersham Pharmacia Biotech, Inc.). The membranes were blocked in 5% nonfat milk in PBS and 0.1% Tween-20 at room temperature. The blots were then incubated with primary antibody: anti-Caspase-3 antibody (1:1000, abcam, Inc.), anti-Bcl-2 antibody (1:1000, Immunoway Biotech, Inc.), anti-Bax antibody (1:1000, abcam, Inc.), anti-Beclin-1 (1:1000, Santa Cruz Biotech, Inc.), anti-LC3 (1:1000, abcam, Inc.), anti-NCX1 (1:1000, abcam, Inc.), anti-Serca2a (1:1000, abcam, Inc.)0 or anti-GAPDH (Santa Cruz Biotech, Inc.). Then the membranes were incubated for 1 h with a secondary antibody (HRP-conjugated anti-rabbit Ig-G, 1:2000). Excess antibody was washed off with TBS-T three times (15 min each) before incubation enhanced chemiluminescent reagent (ECL, R&D Systems Inc., Minneapolis, USA) for 1 min. Subsequently, the membrane was exposed to X-ray film. Immunoreactive bands were detected by the analysis of X-ray films using the software of Image J. The quantity of target proteins is normalized by GAPDH expression.

### Statistical analysis

The SPSS 18.0 software was used for statistical analysis. Data were presented as the mean ± S.E.M. Grouped data were analyzed using a one-way analysis of variance followed by the Student-Newman-Keuls test between each group. A *P* value <0.05 was considered to be statistically significant.

## Results

### Effects of MYQ on cardiac function after I/R injury

To determine the effects of MYQ on cardiac function in rat subject to I/R injury, cardiac function measurements were performed one week or one month after reperfusion. I/R significantly decreases cardiac function compared with the sham control group, by decreasing the systolic arterial pressure (SAP), Pmax, ± dp/dtmax, LVEDP and other parameters (Table 2). One-week treatment with MYQ improves the cardiac function parameters under high dosage; One-month treatment with MYQ significantly ameliorates cardiac function both under low and high dosage.

**Table 2:** Effect of MYQ on cardiac function

	Sham (*n* = 10)	I/R (1 week)			I/R (1 month)		
		I/R (*n* = 10)	Low dose (*n* = 10)	High dose (*n* = 9)	I/R (*n* = 10)	Low dose (*n* = 10)	High dose (*n* = 9)
HR (bpm)	403.8 ± 20.8	354.8 ± 50.8	374.6 ± 26.5	443.6 ± 18.9	310.8 ± 22.4	355.7 ± 34.0	394.0 ± 41.8
RRI (ms)	147.9 ± 6.8	185.8 ± 28.3	163.3 ± 11.7	136.3 ± 6.3	203.7 ± 22.4	179.7 ± 17.0	166.8 ± 29.1
SA P(mmHg)	91.7 ± 3.4	80.8 ± 2.2*	70.2 ± 13.3	68.1 ± 4.7	57.5 ± 2.1*	72.4 ± 9.2†	82.3 ± 3.3
DAP (mmHg)	82.2 ± 2.8	50.0 ± 2.4*	45.9 ± 11.2	54.3 ± 5.5	44.8 ± 1.0*	50.5 ± 8.0	61.7 ± 3.8
MAP (mmHg)	86.8 ± 2.3	61.9 ± 1.8*	55.9 ± 11.7	60.7 ± 4.9	50.8 ± 0.9*	59.0 ± 8.2	70.7 ± 3.6
PP (mmHg)	9.5 ± 3.7	30.7 ± 2.4*	24.3 ± 4.2	13.7 ± 3.2	12.7 ± 2.5	21.9 ± 2.2	20.5 ± 1.1
Pmax (mmHg)	100.5 ± 7.5	87.6 ± 1.8*	82.7 ± 10.5	98.3 ± 6.0†	80.5 ± 2.1*	90.8 ± 6.8†	105.5 ± 3.1†
Pmin (mmHg)	2.8 ± 0.9	5.8 ± 0.4*	5.5 ± 1.9	5.1 ± 3.8	7.8 ± 1.9*	11.0 ± 3.4	2.4 ± 1.9†
Pmean (mmHg)	54.9 ± 2.8	42.8 ± 1.4*	40.5 ± 7.2	49.8 ± 1.5†	37.9 ± 0.9*	41.9 ± 5.0†	51.4 ± 1.7†
Lvedp (mmHg)	15.4 ± 2.3	41.9 ± 14.3*	30.6 ± 13.2	41.0 ± 6.7	19.5 ± 3.7*	18.6 ± 6.4	37.6 ± 3.4
P@dp/dtmax (mmHg)	67.8 ± 8.6	67.0 ± 3.2	64.1 ± 11.6	83.9 ± 5.1	57.3 ± 1.2	64.8 ± 7.3	87.0 ± 3.5
P@-dp/dtmax (mmHg)	69.5 ± 3.8	46.1 ± 1.1	46.7 ± 7.5	49.0 ± 2.6	47.0 ± 2.7	50.2 ± 5.8	50.3 ± 1.8
RPP	33,425 ± 7359	30,482 ± 4066	31,914 ± 5969	43,830 ± 3697	25,151 ± 1953	33,544 ± 5012	42,123 ± 5324
dp/dtmax (mmHg/s)	4943 ± 142	3637 ± 168*	3247 ± 299	3744 ± 323†	3452 ± 240*	4078 ± 643†	4125 ± 146†
-dp/dtmax (mmHg/s)	−4479 ± 155	−3893 ± 149*	−3223 ± 567	−4167 ± 678†	−2753 ± 315*	−3161 ± 487†	4528 ± 358†
At (CFL)(CFU)	102.8 ± 52.1	65.5 ± 108*	40.8 ± 9.9	52.7 ± 11.3	37.9 ± 2.5*	48.3 ± 9.7†	72.9 ± 3.8†
A1 (CFL)(CFU)	9.7 ± 3.5	24.7 ± 9.5*	7.5 ± 0.8	10.7 ± 1.5†	9.0 ± 1.4	13.3 ± 3.1†	13.9 ± 1.2†
A2 (CFL)(CFU)	12.5 ± 2.0	10.5 ± 1.2*	8.6 ± 1.1	9.8 ± 1.5	10.4 ± 1.3*	4.41 ± 2.9†	12.4 ± 0.7†
A3 (CFL)(CFU)	10.4 ± 3.1	12.8 ± 0.7	10.5 ± 2.9	15.8 ± 3.9	7.6 ± 1.1*	10.0 ± 2.0†	18.3 ± 2.0†
A4 (CFL)(CFU)	70.2 ± 49.8	17.5 ± 2.1*	14.2 ± 5.6	16.4 ± 5.7	10.9 ± 1.8*	10.6 ± 2.1	28.4 ± 5.3†
As (CFL)(CFU)	22.2 ± 5.4	35.2 ± 9.1*	16.1 ± 1.9†	20.5 ± 2.8†	19.4 ± 2.6*	27.7 ± 5.9†	26.2 ± 1.9†
Ad (CFL)(CFU)	80.6 ± 50.2	30.3 ± 2.2*	24.7 ± 8.4	32.2 ± 9.5	18.5 ± 1.0*	20.6 ± 4.1	45.7 ± 3.6†
Smax (CRHL)	160.4 ± 49.6	138.0 ± 89.4	214.9 ± 27.3	271.3 ± 43.9	239.4 ± 17.1*	278.2 ± 68.4	263.7 ± 18.2†
Smin (CRHL)(mmHg2/s2)	−202.2 ± 87.0	−274.2 ± 25.9	−248.3 ± 40.0	−389.5 ± 56.9	−240.7 ± 25.6	−350.5 ± 88.0	−392.3 ± 31.1†
Dmax (CRHL)(mmHg2/s)	155.9 ± 66.0	302.3 ± 25.5*	210.1 ± 1.2	303.5 ± 98.8	168.6 ± 16.7	228.6 ± 57.6	348.7 ± 42.1†
Dmin (CRHL)(mmHg2/s2)	−191.9 ± 16.8	−276.1 ± 17.8*	−190.0 ± 43.6	−315.7 ± 55.9	−164.9 ± 18.7*	−211.3 ± 49.1†	−313.8 ± 34.1†

Table 2: cardiac function parameters. HR (Heart rate), RRI (the R-R interval), SAP (Systolic arterial pressure), DAP (Diastolic arterial pressure), MAP (Mean arterial pressure), PP (Pulse pressure), Pmax (the maximum of left ventricular pressure development), Pmin (the minimum of left ventricular pressure development), Pmean (the mean of ventricular pressure development), LVEDP (left ventricular end-diastolic pressure), P@dp/dtmax (the left ventricular pressure corresponding to the rates of maximum positive left ventricular pressure development), P@-dp/dtmax (the left ventricular pressure corresponding to the rates of maximum negative left ventricular pressure development), RPP (rate pressure product), +dp/dtmax (rates of maximum positive left ventricular pressure development), −dp/dtmax (rates of maximum negative left ventricular pressure development), CFL (cardiac force loop), At(CFL) (total area of CFL), A1(CFL) (area of the first CFL), A2(CFL) (area of the second CFL), A3(CFL) (area of the third CFL), A4(CFL) (area of the fourth CFL), As(CFL) (systolic area of CFL), Ad(CFL) (diastolic area of CFL), CRHL (contraction relaxation harmoniousness loop), Smax (CRHL) (the maximum of positive left ventricular systolic pressure of d^2^p/dt^2^), Smin (CRHL) (the maximum of negative left ventricular systolic pressure of d^2^p/dt^2^), Dmax (CRHL) (the maximum of positive left ventricular diastolic pressure of d^2^p/dt^2^),Dmin (CRHL) (the maximum of negative left ventricular diastolic pressure of d^2^p/dt^2^) were measured by a cardiac function analysis system. Values were expressed as mean ± S.E.M. Sham: Sham group; I/R: ischemic/reperfusion group. Low dose: I/R + (1 ml) MYQ treatment group. High dose: I/R + (4 ml) MYQ treatment group. *P < 0.05 compared with sham group. †*P* < 0.05 compared with I/R group

### Effects of MQY on myocardial infarct size after I/R injury

**Fig. 1:**
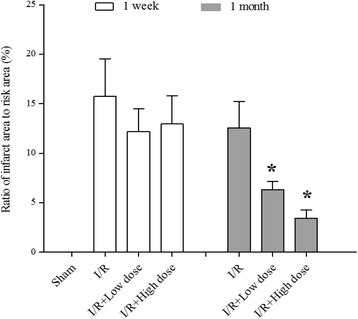
Effects of MYQ on the infarct size caused by occlusion and reperfusion of the left anterior descending coronary artery in anesthetized rat hearts. MYQ was given after two-hours reperfusion (P.O. tid for 1 week or 1 month). The ratio of infarct size to area at risk was measured after one-week or one-month treatment with MYQ. Sham: Sham group (*n* = 10); I/R: ischemic/reperfusion group (*n* = 10). I/R + Low dose: Low dose (1 ml, P.O. tid for one-week or 1 month, *n* = 10 in each group) MYQ treatment group. I/R + High dose: High dose (4 ml, P.O. tid for one-week or 1 month, *n* = 9 in each group) MYQ treatment group. All data were expression as mean ± S.E.M. * *P* < 0.05 compared with I/R group

Cardiomyocyte injury is characterized by myocardial infarct size. To determine whether MQY attenuates I/R-induced cardiomyocyte injury, ratio of infarct size to area-at-risk was calculated. Our data show that ratio of infarct size to area-at risk was still markedly high after one-week or one-month reperfusion. Treatment with MQY for 1 week did not significantly change the ratio, however, treatment for 1 month significantly reduces the ratio (Fig. 1). Data suggest the long-term therapeutic effects of MQY on I/R-induced cardiomyocyte injury.

### Effects of MQY on apoptotic signal pathway in rat I/R model

**Fig. 2:**
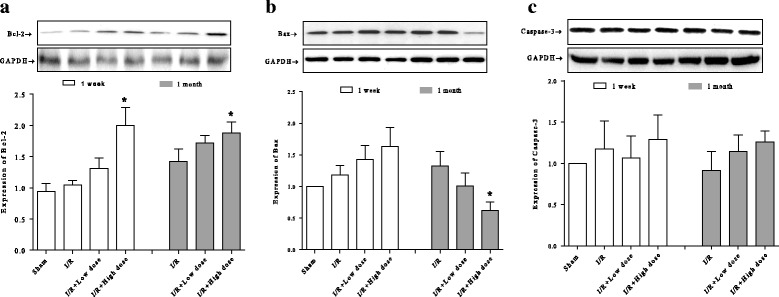
Effects of MYQ on the expression of Bcl-2, Bax, Caspase-3. **a** Expression of the anti-apoptotic protein Bcl-2, upper is the representative blots of Bcl-2 and GAPDH; lower is the densitometric analysis of Bcl-2 expression normalized to GAPDH (*n* = 8). **b** Expression of the pro-apoptotic protein Bax, upper is the representative blots of Bax and GAPDH; lower is the densitometric analysis of Bax expression normalized to GAPDH (*n* = 8). **c** Expression of the caspase-3, upper is the representative blots of caspase-3 and GAPDH; lower is the densitometric analysis of caspase-3 expression normalized to GAPDH (*n* = 8). Sham: Sham group; I/R: ischemic/reperfusion group. I/R + Low dose: Low dose (1 ml, P.O. tid for one-week or 1 month) MYQ treatment group. I/R + High dose: High dose (4 ml, P.O. tid for one-week or 1 month) MYQ treatment group. All data were expression as mean ± S.E.M. * *P* < 0.05 compared with I/R group

Bcl-2 and Bax genes are reported to play a crucial role in cell survival or death after apoptotic stimuli [32]. Caspase-3 is also an important component of the apoptotic pathway [33]. The effects of MQY on Bcl-2, Bax, and caspase-3 expression in myocardial tissue were analyzed by western blot. I/R for one-week or 1 month did not show any marked effects on the expression of these proteins compared with sham group. However, anti-apoptotic protein, Bcl-2 was significantly increases in high doses groups of MQY treatment for one-week or one-month compared with I/R group (Fig. 2a). In addition, expression of pro-apoptotic protein, Bax, was markedly reduced in high dose group of MQY treatment for one-month compared with I/R group (Fig. 2b). Levels of caspase-3 were not significantly different among all groups (Fig. 2c). These results indicate that MQY may exerts its protective effects via parts of apoptotic signal pathway.

### Effects of MQY on Ca^2+^ handling proteins expression in rat I/R model

**Fig. 3:**
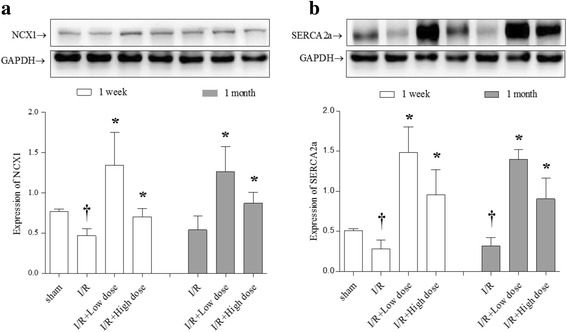
Effects of MYQ on the expression of NCX1 and SERCA2a. **a** Expression of SERCA2a, upper is the representative blots of NCX1 and GAPDH; lower is the densitometric analysis of NCX1 expression normalized to GAPDH (*n* = 8). **b** Expression of SERCA2a, upper is the representative blots of SERCA2a and GAPDH; lower is the densitometric analysis of SERCA2a expression normalized to GAPDH (*n* = 8). Sham: Sham group; I/R: ischemic/reperfusion group. I/R + Low dose: Low dose (1 ml, P.O. tid for one-week or 1 month) MYQ treatment group. I/R + High dose: High dose (4 ml, P.O. tid for one-week or 1 month) MYQ treatment group. All data were expression as mean ± S.E.M. † *P* < 0.05 compared with sham group, * *P* < 0.05 compared with I/R group

It is well-known that I/R could influence the Ca^2+^ regulatory protein expressions [34]. Our results show that one-week after I/R, both expressions of NCX1 and Serca2a were significantly reduced compared with sham group. Treatment with MQY for one-week could reverse these reductions (both of low and high dose). In addition, after one-month I/R, expression of NCX1 were still low, but not significantly different with sham group, and MQY treatment also markedly increased NCX1 expressions (both of low and high dose). Furthermore, 1 month after I/R, expression of Serca2a was still markedly lower compared with sham group, and MQY treatment also significantly increased the Serca2a expression compared with I/R group (Fig. 3). There results suggest that MQY could up-regulate Ca^2+^ handling protein expressions therefore ameliorate intracellular Ca^2+^ overload in response to cardiac I/R.

### Effects of MQY on autophagic signal pathway in rat I/R model

**Fig. 4:**
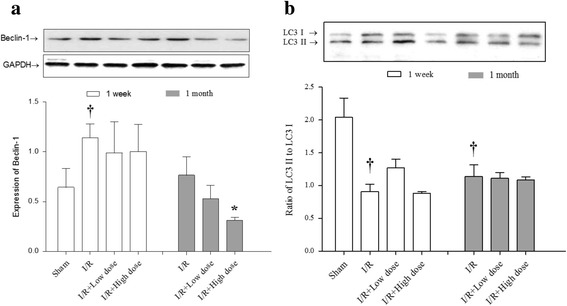
Effects of MYQ on the expression of Beclin-1 and LC3. **a** Expression of Beclin-1, upper is the representative blots of Beclin-1 and GAPDH; lower is the densitometric analysis of Beclin-1 expression normalized to GAPDH (*n* = 8). **b** Expression of LC3, upper is the representative blots of LC3 I and LC3 II; lower is the densitometric analysis of the ratio of LC3 II to LC3 I (*n* = 8). Sham: Sham group; I/R: ischemic/reperfusion group. I/R + Low dose: Low dose (1 ml, P.O. tid for one-week or 1 month) MYQ treatment group. I/R + High dose: High dose (4 ml, P.O. tid for one-week or 1 month) MYQ treatment group. All data were expression as mean ± S.E.M. † *P* < 0.05 compared with sham group

Although it is still controversial about role of autophagy in response to reperfusion, existing evidence demonstrated that reperfusion induces upregulation of Beclin-1, which further impairs autophagosome processing, culminating in increased reactive oxygen species generation, mitochondrial permeabilization, and cardiomyocyte death [35]. Our investigation found that expression of Beclin-1 was higher after I/R for 1 week than that of sham group, which consists with previous observation. Interestingly, treatment with MQY for 1 week showed no marked increase of Beclin-1 expression (both in low and high dose groups) compared with control group. Importantly, although 1 month after I/R, expression of Beclin-1 return to normal level, treatment with high dose of MQY significantly reduced the expression of Beclin-1 compared with I/R group (Fig. 4a). These data suggest that MQY could regulate Beclin-1 expression, which may alleviate impaired autophagosome processing and cardiomyocyte death.

LC3 is also an important marker for autophagy levels [36]. In the present study, we found that I/R reduced LC3-II expression both in one-week and one-month group compared with sham group, strongly suggest I/R could impair autophagic flux. However, treatment with MQY had no effect on reduction of LC3-II expression in low or high dose groups for one-week or one-month (Fig. 4b). These results suggest that MQY might exert protective effects through specific regulation of autophagic signal pathway.

## Discussion

In the present study, our data clearly demonstrate that MYQ could improve cardiac function and reduce myocardial injury in response to long term I/R injury. The involved mechanism may be through regulation of cell apoptosis cascades (increase of Bcl2 and decrease Bax expressions), increasing Ca^2+^ handling proteins (increase of NCX1 and Serca2a expression), and regulating autophagic proteins signal pathways (decrease of Beclin-1 expression).

MYQ used in the present study is composed of five herbs, each having been widely used in Traditional Chinese Medicine for various diseases. *Milkvetch Root* has been ascribed to be an important herb to improve Qi [7]. BuYang HuanWu decotion, composed of *Milkvetch Root* and other herbs, has been reported to has protective effects on myocardial ischemia induced by isoproterenol in rats [37]. *Milkvetch Root* also has been reported to has specific effects on Ca^2+^ handling both in cellular level [10, 11] and whole body level [12]. Recently, protective effects of *Milkvetch Root* against endothelial dysfunction in hypertrophic rats induced by isoproterenol also has been reported [38]. Besides the effects of *Milkvetch Root* on cardiovascular diseases, its effects on other diseases also have been reported, such as malignant plural effusion [14], diabetes [39, 40], and cancer [41]. Although *Semen Pharbitidis* is reported to has toxicology effect after long term administration [42], its therapeutic effect on relieving constipation by purgation, dispersing phlegm and washing excessive fluid is also well investigated, and is widely used for the treatment of edema, ascites, hydroncus, simple obesity, lung fever, tumor and ardent fever [43–45]. Extraction of *Semen Pharbitidis* also shows antioxidant effect [46]. *Cinnamom Twig* has excellent antihyperglycemic, antioxidant, protein tyrosine phosphatase 1B inhibitory activities [17], and anti-inflammatory activities [47]. A population-based study also showed that *Cinnamom Twig* has sedative and anti-inflammatory effect for the treatment of endometriosis-related symptomatic discomfort [15]. *Fructus Amomi* is used to treat stomach disorders, pulmonary diseases and liver complaints [48, 49], which also has antioxidant activity [50]. *White Peony Root* has been reported to promote the recovery of bone marrow hemopoietic function in a myelosuppressed mouse model [51], and its therapeutic effect on radiation-induced esophageal toxicity is also has been observed [52, 53]. Based on above-mentioned literatures, composition of MYQ includes the herbs for improving cardiovascular and digestive function, and regulating immune system, which is accordance with the core concept of Wu Meng Therapy: treatment of cardiovascular diseases combined with circulatory and digestive systems. In the present study we firstly explore the therapeutic effects of MYQ in I/R induced cardiac injury, secondly, we investigate the possible mechanisms involved in.

It is well-known that reperfusion of heart results in further damage of heart tissue, and induces reduction of heart function [54, 55]. Our investigation clearly shows that there is significant reduction of cardiac function after one-week and one-month reperfusion, which is concordance with previous report [55]. However, MQY treatment could improve cardiac function after I/R one-week (high dose) and one-month (both low dose and high dose, Table 2). These results strongly reveal the therapeutic effect of MQY in response to I/R induced reduction of cardiac function. In addition, our results also show that tissue injury is ameliorated after treatment of MQY for one-month, although one-week treatment does not show any significant improvement. It should be noted that dosages, especially the high dose, used in the present study are well-designed to investigated the mechanisms involved in. Also, it should be mentioned that time of MQY treatment is more similar for treatment of clinical patients with cardiac infarction patients, which might provide more precise evidence for clinical application.

Traditionally, it has been well recognized that cellular responses to I/R is highly related to the activation of apoptotic pathways and various strategies were explored to suppress the activation of apoptosis [56, 57]. Although our present data does not show any markedly effects of MQY on apoptosis signal pathway with low dose, however, high dose of MQY shows significant effect on regulation of anti-apoptotic protein Bcl-2 and pro-apoptotic protein Bax, which implies that MQY exerts its protective effect may be through regulation of apoptotic signal pathway. Interestingly, expression of caspase-3, which is best known for its role in the execution phase of apoptosis, is not different after one-week or one-month reperfusion compared with control group. We speculate that caspase signal pathways only play pivotal role in short term of injurious stimuli, this issue still needs further investigation. Our results also show that MQY has no effects on regulation of caspase-3 expression even after only term treatment.

It is well documented that the cellular Ca^2+^ concentration is highly related to cellular necrosis in response to I/R injury, and the mechanism for this process involves the restoration of oxygen, which enables ATP-generating capabilities to quickly recover ATP levels and the mitochondrial membrane potential. These changes regenerate the required ion gradient for more Ca^2+^ entry into the mitochondria, which causes the long-lasting opening of the MPTP, which is regulated by cyclophilin D, and mitochondrial swelling ultimately leads to cellular necrosis [58]. Thereafter, the increased ability of Ca^2+^ handling apparently reduces I/R-induced mitochondrial swelling and subsequently cellular necrosis. Our present study clearly demonstrated that Ca^2+^ handling proteins NCX1 and Serca2a are markedly down-regulated after I/R for one-week or one-month. Treatment with MQY significantly increase expressions of NCX1 and Serca2a, suggesting therapeutic effect of MQY may also be via regulation of Ca^2+^ handling proteins, which is concordance with previous observation [10–12].

Under stress conditions, autophagy is activated either to meet the increased requirements for repair and detoxication as a result of an exposure to various damaging factors or to produce energy and deliver building blocks for anabolic processes under starvation. To date, data consistently report that autophagy is protective under conditions of mild-to-moderate ischemia; however, the upregulation of autophagy can be either beneficial [59] or detrimental [35]. In the present study, we observed that Beclin-1 is up-regulated after one-week reperfusion, which is concordance with previous study that demonstrated reactive oxygen species mediating decline in lysosome-associated membrane protein-2 and upregulation of Beclin-1, thereafter impairs autophagosome clearance [35]. Our results show that MQY treatment for one-week do not significantly increase Beclin-1 and treatment for one-month with high dose markedly reduced Beclin-1 expression, suggesting MQY plays therapeutic effects may be through down-regulation of Beclin-1. However, we also observed that LC3-II expression is markedly down-regulated after I/R one-week and one-month. The reason for LC3-II down-regulation may be due to negative feedback in response to impaired autophaosome clearance after I/R. This issue also needs further investigation. Our present observation also shows that MQY had no effect on reduction of LC3-II expression.

## Conclusions

In conclusion, our present observation demonstrates that MYQ have potential therapeutic effects on I/R-induced cardiac injury, which may be through regulation of apoptotic proteins, cytosolic Ca^2+^ handling proteins and autophagic proteins signal pathways.

## Abbreviations

IR: Ischemia-reperfusion
MYQ: Modified Yi Qi decoction
NCX1: Sodium calcium exchanger
Serca2a: Sarcoplasmic reticulum Ca2 + −ATPase

## References

[1] Mozaffarian D, Benjamin EJ, Go AS, Arnett DK, Blaha MJ, Cushman M, de Ferranti S, Despres JP, Fullerton HJ, Howard VJ, Huffman MD, Judd SE, Kissela BM, Lackland DT, Lichtman JH, Lisabeth LD, Liu S, Mackey RH, Matchar DB, McGuire DK, Mohler ER, 3rd, Moy CS, Muntner P, Mussolino ME, Nasir K, Neumar RW, Nichol G, Palaniappan L, Pandey DK, Reeves MJ, Rodriguez CJ, Sorlie PD, Stein J, Towfighi A, Turan TN, Virani SS, Willey JZ, Woo D, Yeh RW, Turner MB, American Heart Association Statistics C, Stroke Statistics S: Heart disease and stroke statistics--2015 update: a report from the American Heart Association. Circulation 2015, 131:e29–322. 10.1161/CIR.0000000000000152 25520374

[2] Lopez AD, Murray CC (1998). The global burden of disease, 1990-2020. Nat Med.

[3] Hausenloy DJ, Yellon DM (2013). Myocardial ischemia-reperfusion injury: a neglected therapeutic target. J Clin Invest.

[4] Murphy E, Steenbergen C (2008). Mechanisms underlying acute protection from cardiac ischemia-reperfusion injury. Physiol Rev.

[5] Jordan JE, Zhao ZQ, Vinten-Johansen J (1999). The role of neutrophils in myocardial ischemia-reperfusion injury. Cardiovasc Res.

[6] Fauconnier J, Roberge S, Saint N, Lacampagne A (2013). Type 2 ryanodine receptor: a novel therapeutic target in myocardial ischemia/reperfusion. Pharmacol Ther.

[7] Liu Q, Li J, Wang J, Li J, Janicki JS, Fan D (2013). Effects and mechanisms of chinese herbal medicine in ameliorating myocardial ischemia-reperfusion injury. Evidence-based Complementary and Alternative Medicine: eCAM.

[8] Zhao FD, Dong JC, Xie JY: [Effects of Chinese herbs for replenishing shen and strengthening qi on some indexes of neuro-endocrino-immune network in asthmatic rats]. Zhongguo Zhong xi yi jie he za zhi Zhongguo Zhongxiyi jiehe zazhi = Chinese journal of integrated traditional and Western medicine/Zhongguo Zhong xi yi jie he xue hui, Zhongguo Zhong yi yan jiu yuan zhu ban 2007, 27:715–9. 17879536

[9] Jiang D, Wang X, Su Q, Jiang S, Yuan F, Zhang C (2015). Milkvetch root improves immune function in patients with acute exacerbation of COPD. Biomed Mater Eng.

[10] Meng D, Chen XJ, Bian YY, Li P, Yang D, Zhang JN (2005). Effect of astragalosides on intracellular calcium overload in cultured cardiac myocytes of neonatal rats. The American Journal of Chinese Medicine.

[11] Xu XL, Chen XJ, Ji H, Li P, Bian YY, Yang D (2008). Astragaloside IV improved intracellular calcium handling in hypoxia-reoxygenated cardiomyocytes via the sarcoplasmic reticulum ca-ATPase. Pharmacology.

[12] Wang Y, Ji Y, Xing Y, Li X, Gao X (2012). Astragalosides rescue both cardiac function and sarcoplasmic reticulum ca(2)(+) transport in rats with chronic heart failure. Phytotherapy Research: PTR.

[13] Li JH, Du GJ, Liu WJ, Liu YH, Zhao B, Li H (2014). [study on anti-tumor and anti-metastasis mechanism of alcohol extracts from pharbitidis semen against Lewis lung cancer]. Zhongguo Zhong yao za zhi = Zhongguo zhongyao zazhi =. China Journal of Chinese Materia Medica.

[14] Feize W, Meng L, Yanni L, Yuan L, Liqun J, Tong L, et al. A randomized controlled study to observe the efficacy of external treatment with a traditional Chinese medicine herbal ointment on malignant plural effusion: outcome report and design review. Integrative Cancer Therapies. 2016; 10.1177/1534735416660193 PMC5739134 27431570

[15] Fang RC, Tsai YT, Lai JN, Yeh CH, Wu CT (2012). The traditional chinese medicine prescription pattern of endometriosis patients in taiwan: a population-based study. Evidence-based Complementary and Alternative Medicine: eCAM.

[16] Yen HR, Chen YY, Huang TP, Chang TT, Tsao JY, Chen BC (2015). Prescription patterns of Chinese herbal products for patients with uterine fibroid in Taiwan: a nationwide population-based study. J Ethnopharmacol.

[17] Lin GM, Chen YH, Yen PL, Chang ST (2016). Antihyperglycemic and antioxidant activities of twig extract from Cinnamomum osmophloeum. Journal of Traditional and Complementary Medicine.

[18] Feng S, Song L, Liu Y, Lai F, Zuo G, He G (2013). Hypoglycemic activities of commonly-used traditional Chinese herbs. The American journal of Chinese Medicine.

[19] Guo DJ, Cheng HL, Chan SW, Yu PH (2008). Antioxidative activities and the total phenolic contents of tonic Chinese medicinal herbs. Inflammopharmacology.

[20] Choi HG, Je IG, Kim GJ, Choi H, Kim SH, Kim JA (2015). Anti-allergic inflammatory activities of compounds of amomi fructus. Nat Prod Commun.

[21] Yeh LL, Liu JY, Liu YS, Lin KS, Tsai TF, Wang LH (2009). Anemia-related hemogram, uterine artery pulsatility index, and blood pressure for the effects of four-agents-decoction (Si Wu tang) in the treatment of primary dysmenorrhea. J Altern Complement Med.

[22] Wang Z, Shen L, Li X, Shu X, Shan B, Zhang L (2013). Painrelieving effect of a compound isolated from white peony root oral liquid on acute radiationinduced esophagitis. Mol Med Rep.

[23] Jin X, Zhang Y, Li Q, Zhao J (2013). Mechanisms underlying the beneficial effects of Kaiyu granule for depression. Neural Regen Res.

[24] Feng L, Liu WK, Deng L, Tian JX, Tong XL: Clinical efficacy of aconitum-containing traditional Chinese medicine for diabetic peripheral neuropathic pain. The American Journal of Chinese Medicine 2014, 42:109–117. 10.1142/S0192415X14500074 24467538

[25] Yin HQ, Wang B, Zhang JD, Lin HQ, Qiao Y, Wang R (2008). Effect of traditional Chinese medicine Shu-Mai-tang on attenuating TNFalpha-induced myocardial fibrosis in myocardial ischemia rats. J Ethnopharmacol.

[26] Mak DH, Chiu PY, Dong TT, Tsim KW, Ko KM (2006). Dang-Gui Buxue tang produces a more potent cardioprotective effect than its component herb extracts and enhances glutathione status in rat heart mitochondria and erythrocytes. Phytotherapy Research: PTR.

[27] Yin H, Zhang J, Lin H, Qiao Y, Wang R, Lu H (2009). Effect of traditional Chinese medicine Shu-mai-tang on angiogenesis, arteriogenesis and cardiac function in rats with myocardial ischemia. Phytotherapy Research: PTR.

[28] Wang XY, Qin F, Huang X, Zhang XY, Ren P, Zhao HW (2009). [effects of Dan-Chuan-Hong decoction on myocardial apoptosis of acute myocardial ischemia in rats]. Zhong yao cai = Zhongyaocai =. Journal of Chinese Medicinal Materials.

[29] Chiu PY, Wong SM, Leung HY, Leong PK, Chen N, Zhou L (2011). Acute treatment with Danshen-Gegen decoction protects the myocardium against ischemia/reperfusion injury via the redox-sensitive PKCvarepsilon/mK(ATP) pathway in rats. Phytomedicine: international journal of phytotherapy and phytopharmacology.

[30] Chiu PY, Leung HY, Leong PK, Chen N, Zhou L, Zuo Z (2012). Danshen-Gegen decoction protects against hypoxia/reoxygenation-induced apoptosis by inhibiting mitochondrial permeability transition via the redox-sensitive ERK/Nrf2 and PKCepsilon/mKATP pathways in H9c2 cardiomyocytes. Phytomedicine: International Journal of Phytotherapy and Phytopharmacology.

[31] Zhang GX, Kimura S, Murao K, Obata K, Matsuyoshi H, Takaki M (2010). Inhibition of cytochrome c release by 10-N-nonyl acridine orange, a cardiolipin-specific dye, during myocardial ischemia-reperfusion in the rat. Am J Phys Heart Circ Phys.

[32] Scarfo L, Ghia P (2013). Reprogramming cell death: BCL2 family inhibition in hematological malignancies. Immunol Lett.

[33] Mirzayans R, Andrais B, Kumar P, Murray D. The growing complexity of cancer cell response to DNA-damaging agents: caspase 3 mediates cell death or survival? Int J Mol Sci. 2016;17 10.3390/ijms17050708 PMC4881530 27187358

[34] La Rovere RM, Roest G, Bultynck G, Parys JB. Intracellular Ca2+ signaling and Ca2+ microdomains in the control of cell survival, apoptosis and autophagy. Cell Calcium. 2016; 10.1016/j.ceca.2016.04.005 27157108

[35] Ma X, Liu H, Foyil SR, Godar RJ, Weinheimer CJ, Hill JA (2012). Impaired autophagosome clearance contributes to cardiomyocyte death in ischemia/reperfusion injury. Circulation.

[36] Chiong M, Wang ZV, Pedrozo Z, Cao DJ, Troncoso R, Ibacache M (2011). Cardiomyocyte death: mechanisms and translational implications. Cell Death Dis.

[37] Liu Y, Lin R, Zhang H, Zhang JY, Ji QL, Yang YJ: [Protective effect of Buyanghuanwu Decoction on myocardial ischemia induced by isoproterenol in rats]. Zhong yao cai = Zhongyaocai = Journal of Chinese medicinal materials 2009, 32:380–3. 19565716

[38] Han R, Tang F, Lu M, Xu C, Hu J, Mei M (2016). Protective effects of Astragalus polysaccharides against endothelial dysfunction in hypertrophic rats induced by isoproterenol. Int Immunopharmacol.

[39] Dun C, Liu J, Qiu F, Wu X, Wang Y, Zhao Y (2016). Effects of Astragalus polysaccharides on memory impairment in a diabetic rat model. Neuropsychiatr Dis Treat.

[40] Yi YE, Li SY, Nie YN, Jia DX, Zhang ZH, Wang YF (2016). Effect of astragalus injection on renal tubular epithelial transdifferentiation in type 2 diabetic mice. BMC Complement Altern Med.

[41] Wang SF, Wang Q, Jiao LJ, Huang YL, Garfield D, Zhang J (2016). Astragalus-containing traditional Chinese medicine, with and without prescription based on syndrome differentiation, combined with chemotherapy for advanced non-small-cell lung cancer: a systemic review and meta-analysis. Curr Oncol.

[42] Ma C, Bi K, Zhang M, Su D, Fan X, Ji W (2010). Toxicology effects of morning glory seed in rat: a metabonomic method for profiling of urine metabolic changes. J Ethnopharmacol.

[43] Tsai JC, Tsai S, Chang WC (2004). Comparison of two Chinese medical herbs, Huangbai and Qianniuzi, on influence of short circuit current across the rat intestinal epithelia. J Ethnopharmacol.

[44] Lee TH, Choi JJ, Kim DH, Choi S, Lee KR, Son M (2008). Gastroprokinetic effects of DA-9701, a new prokinetic agent formulated with Pharbitis semen and corydalis tuber. Phytomedicine: International Journal of Phytotherapy and Phytopharmacology.

[45] Kim KH, Ha SK, Choi SU, Kim SY, Lee KR (2011). Bioactive phenolic constituents from the seeds of Pharbitis nil. Chemical & Pharmaceutical Bulletin.

[46] Wang Q, Sun Y, Yang B, Wang Z, Liu Y, Cao Q (2014). Optimization of polysaccharides extraction from seeds of Pharbitis nil and its anti-oxidant activity. Carbohydr Polym.

[47] Tung YT, Chua MT, Wang SY, Chang ST (2008). Anti-inflammation activities of essential oil and its constituents from indigenous cinnamon (Cinnamomum osmophloeum) twigs. Bioresour Technol.

[48] Kumar G, Chauhan B, Ali M (2014). Isolation and identification of new phytoconstituents from the fruit extract of Amomum Subulatum Roxb. Nat Prod Res.

[49] Jafri MA (2001). Farah, Javed K, Singh S: evaluation of the gastric antiulcerogenic effect of large cardamom (fruits of Amomum Subulatum Roxb). J Ethnopharmacol.

[50] Wang JH, Shin JW, Choi MK, Kim HG, Son CG (2011). An herbal fruit, Amomum Xanthoides, ameliorates thioacetamide-induced hepatic fibrosis in rat via antioxidative system. J Ethnopharmacol.

[51] Zhu Y, Wang L, Yang Z, Wang J, Li W, Zhou J (2016). Hematopoietic effects of Paeoniflorin and Albiflorin on radiotherapy-induced myelosuppression mice. Evidence-based Complementary and Alternative Medicine.

[52] Shen L, Li X, Shan B, Zhang L, Gong Y, Dong Z (2013). Therapeutic effect of compound of white Peony root oral liquids on radiation-induced esophageal toxicity via the expression of EGF and TGF-beta1. Biomedical Reports.

[53] Wang Z, Shen L, Wang J, Shan B, Zhang L, Lu F (2013). Immunostimulatory effect of a composition isolated from white peony root oral liquid in the treatment of radiation-induced esophagitis. Experimental and Therapeutic Medicine.

[54] Hausenloy DJ, Yellon DM (2016). Ischaemic conditioning and reperfusion injury. Nat Rev Cardiol.

[55] Bulluck H, Yellon DM, Hausenloy DJ (2016). Reducing myocardial infarct size: challenges and future opportunities. Heart.

[56] Yellon DM, Hausenloy DJ (2007). Myocardial reperfusion injury. N Engl J Med.

[57] Bagai A, Dangas GD, Stone GW, Granger CB (2014). Reperfusion strategies in acute coronary syndromes. Circ Res.

[58] Whelan RS, Kaplinskiy V, Kitsis RN (2010). Cell death in the pathogenesis of heart disease: mechanisms and significance. Annu Rev Physiol.

[59] Hamacher-Brady A, Brady NR, Gottlieb RA (2006). Enhancing macroautophagy protects against ischemia/reperfusion injury in cardiac myocytes. J Biol Chem.

